# Variance component testing for identifying differentially expressed genes in RNA-seq data

**DOI:** 10.7717/peerj.3797

**Published:** 2017-09-08

**Authors:** Sheng Yang, Fang Shao, Weiwei Duan, Yang Zhao, Feng Chen

**Affiliations:** Department of Biostatistics, School of Public Health, Nanjing Medical University, China

**Keywords:** RNA-seq, Differentially expressed (DE), Generalized mixed linear model (GLMM), Variance component test (VCT)

## Abstract

RNA sequencing (RNA-Seq) enables the measurement and comparison of gene expression with isoform-level quantification. Differences in the effect of each isoform may make traditional methods, which aggregate isoforms, ineffective. Here, we introduce a variance component-based test that can jointly test multiple isoforms of one gene to identify differentially expressed (DE) genes, especially those with isoforms that have differential effects. We model isoform-level expression data from RNA-Seq using a negative binomial distribution and consider the baseline abundance of isoforms and their effects as two random terms. Our approach tests the global null hypothesis of no difference in any of the isoforms. The null distribution of the derived score statistic is investigated using empirical and theoretical methods. The results of simulations suggest that the performance of the proposed set test is superior to that of traditional algorithms and almost reaches optimal power when the variance of covariates is large. This method is also applied to analyze real data. Our algorithm, as a supplement to traditional algorithms, is superior at selecting DE genes with sparse or opposite effects for isoforms.

## Introduction

The availability of massively parallel transcriptome sequencing technology has improved our understanding of the central dogma processes of transcription and translation and the molecular mechanisms of complex diseases (Koboldt et al., 2013; Modelska, Quattrone & Re, 2015; Wang, Gerstein & Snyder, 2009). RNA sequencing (RNA-Seq) has shown that coding genes are stochastically spliced into different transcripts (called isoforms), including partial exons or selected exons (Kalsotra & Cooper, 2011; Kanitz et al., 2015; Oshlack, Robinson & Young, 2010; Pan et al., 2008). This process is referred to as alternative splicing (AS). The different sequence characteristics of isoforms may exhibit different expression patterns, and these differences further influence the translation of proteins and affect cellular phenotypes (Li et al., 2014).

The elementary problem of transcriptomic data analysis is accurate identification of differentially expressed (DE) genes (Garber et al., 2011). Emerging software packages and pipelines for such analyses have been designed and developed, including methods that rely on gene-level measurement, such as DESeq, edgeR and the two-stage passion model (TSPM), and others based on isoform-level measurements, such as Cuffdiff2, IsoDE and EBSeq (Al Seesi et al., 2014; Anders & Huber, 2010; Lehmann et al., 2011; Li & Tibshirani, 2013; Robinson, McCarthy & Smyth, 2010; Trapnell et al., 2013). The gene-level quantification methods assume that the sum of isoform expression levels constitutes the expression of one gene, which may ignore the variability of different transcripts (Trapnell et al., 2013; Trapnell et al., 2010). Thus, the development of a method for considering isoform-level measurements is of increasing importance. For example, considering both the splicing structure and expression levels of isoforms, Cuffdiff2 uses the beta negative binomial distribution to define DE isoforms (Trapnell et al., 2013). Unfortunately, testing for individual isoforms separately neglects the relationships between each isoform (Dialsingh, Austin & Altman, 2015).

Currently, the idea of a set test, which supposes that some related variables form one set and tests the set, likely overcomes the drawbacks of gene-level measurement methods and the disadvantage of testing isoforms individually. The theoretical basis is that the score statistic of the variance component in a generalized linear mixed model (GLMM) follows the mixture chi-squared distribution with the null hypothesis (Lin, 1997). The sequencing kernel association test (SKAT), a popular algorithm in genome-wide association studies (GWASs), assumes that the effects of each locus are in the same functional region as random effects and uses the score statistics to test the set (Wu et al., 2011). Since the estimation process is under the null hypothesis, this method reduces computation time. The test for the effect of gene set (TEGS), which regresses gene expression against phenotypes, is widely used to select DE genes in a microarray platform. The random effect and the residual error of the model are due to the disease effect and the correlation between genes, respectively (Huang & Lin, 2013). The set test is effectively applied to analyze other types of high-dimensional genomic data (Ionita-Laza et al., 2013; Wu et al., 2016).

Considering the disadvantages of traditional algorithms in selecting heterogeneous genes and the accessibility of the set test, we apply the idea of the set test to identify DE genes in isoform-level measurements. This study involved three parts. First, the relationship between the expression level of one gene and the phenotype is formulated as a GLMM with the assumption of a Poisson and negative binomial (NB) distribution. Second, we study the empirical and theoretical distributions of score statistics and measure their statistical performance for different parameter settings. Third, we analyze level 3 mRNA-Seq data on lung squamous cell carcinoma (LUSC) from The Cancer Genome Atlas (TCGA). Comparisons with traditional algorithms are described in the ‘Simulations’ and ‘Real data analysis’.

## Methods

### Model

Assume that there are *N*subjects in the studied sample, and suppose that for all individuals there is one sequenced gene with *p* isoforms. For the *i*th individual, *Y*_*i*1_, *Y*_*i*2_, …, *Y*_*ip*_ denotes the RNA-seq data of one gene. The vector **Y** is assumed as a Poisson or NB distribution. With the assumption of independent samples, *x*_*i*_ is the covariate of individual *i*. For example, its value is 0 or 1 in a case-control study. We assume *α*_*j*_ to be a random term that follows a normal distribution. Its variance represents the heterogeneity of the baseline abundance of the isoforms. This distinct assumption is the difference between gene-level measurement methods and isoform-level measurement methods. The disease effect is also defined as a random effect. Based on the above assumptions, a GLMM of two random terms is constructed as follows:

\begin{eqnarray*}g \left( E \left( {Y}_{ij} \right) \right) =\mu +{\alpha }_{j}+{\beta }_{j}{x}_{i}, i=1,\ldots ,N \text{and} j=1,\ldots ,p \end{eqnarray*}

where $g \left( . \right) $ is a monotonic differentiable link function, such as $g \left( x \right) =\log \left( x \right) $ for the Poisson regression. If *x*_*i*_ is equal to zero in a case-control study, *μ* + *α*_*j*_ indicates the expression of the *j*th isoform in the control group, and *β*_*j*_ indicates the disease effect.

The matrix form is written as follows:

\begin{eqnarray*}g \left( E \left( \mathbf{Y } \right) \right) =\mu +\mathbf{K}\alpha +\mathbf{X}\beta \end{eqnarray*}

$\mathbf{Y }={ \left( {\mathbf{Y }}_{1}^{T},{\mathbf{Y }}_{2}^{T},\ldots ,{\mathbf{Y }}_{N}^{T} \right) }^{T}$ is an *N* × *p* vector consisting of *N***Y**_*i*_ vectors (**Y**_*i*_ = (*Y*_*i*1_, *Y*_*i*2_, …, *Y*_*ip*_)^*T*^). *μ* is also an *N* × *p* vector, in which all elements are $\mu \cdot \mathbf{K}={ \left( {\mathbf{I}}_{p},\ldots ,{\mathbf{I}}_{p} \right) }^{T}$ is an *Np* × *p* matrix in which **I**_*p*_ is the *p*-dimensional identity matrix, $\alpha ={ \left( {\alpha }_{1},{\alpha }_{2},\ldots ,{\alpha }_{p} \right) }^{T}$, $\mathbf{X}={ \left( {x}_{1}{\mathbf{I}}_{p},{x}_{2}{\mathbf{I}}_{p},\ldots ,{x}_{N}{\mathbf{I}}_{p} \right) }^{T}$, and $\beta ={ \left( {\beta }_{1},{\beta }_{2},\ldots ,{\beta }_{p} \right) }^{T}$.

### Theoretical null distributions of statistics

In fact, the test of the disease effect is performed to test the variance component of the second random effect. As the basic assumption of mixed models, the third term in the above equation follows a normal distribution ($\beta \sim N \left( 0, {\tau }^{2}{\mathbf{I}}_{p} \right) $) (Gad & El Kholy, 2012). Therefore, the test of *β* equals the test of its variance component *τ*. The final null hypothesis (*H*_0_) of the model is written as *τ* = 0. The idea of a score test is used to test *τ*. Based on the theory of score statistics in a GLMM and the special assumption of this model, the null distribution and parameter estimation of the statistic *U* is as follows:

First, the matrix form of the first order derivate of the log-likelihood is as follows:

\begin{eqnarray*} \frac{\partial l \left( \alpha ,\tau \right) }{\partial \tau } = \frac{1}{2} \left( { \left( \mathbf{Y }-\mu -\mathbf{K}\alpha \right) }^{T}{\Delta }^{-\mathbf{1}}\mathbf{WX}{\mathbf{X}}^{T}{\Delta }^{-\mathbf{1}}\mathbf{W} \left( \mathbf{Y }-\mu -\mathbf{K}\alpha \right) -tr \left( {\mathbf{W}}_{0}\mathbf{X}{\mathbf{X}}^{T} \right) \right) \end{eqnarray*}

where Δ^−**1**^, **W** and **W**_0_ are block diagonal matrixes, and $\mathbf{W}=E \left( {\mathbf{W}}_{0} \right) $. Each block of the three matrices is a diagonal matrix whose diagonal elements are similar. Assuming ${\gamma }_{i}=E \left( {y}_{i} \right) $, their diagonal elements are ${\delta }_{i}=1/{g}^{{^{\prime}}} \left( {\gamma }_{i} \right) ,{w}_{i}=V{ \left( {\gamma }_{i} \right) }^{-1}{\delta }_{i}^{2}\text{and}{w}_{0i}={w}_{i}+{e}_{i} \left( {y}_{i}-{\gamma }_{i} \right) $, where ${e}_{i}= \left( {V}^{{^{\prime}}} \left( {\gamma }_{i} \right) {g}^{{^{\prime}}} \left( {\gamma }_{i} \right) +V \left( {\gamma }_{i} \right) {g}^{{^{\prime\prime}}} \left( {\gamma }_{i} \right) \right) / \left( {V}^{2} \left( {\gamma }_{i} \right) { \left( {g}^{{^{\prime}}} \left( {\gamma }_{i} \right) \right) }^{3} \right) $. With different variances and the same link function, Δ^−**1**^ of Poisson and NB distribution is the same, but **W** and **W**_0_ are different. For the Poisson assumption, the product of Δ^−**1**^ and **W** is an identity matrix, and **W** = **W**_0_. For the NB assumption, we take the product of Δ^−**1**^ and **W** as Φ, which has diagonal elements of $1/ \left( 1+\phi {\gamma }_{i} \right) $ . *ϕ* is the scale parameter of an NB. The diagonal elements of the block of **W**_0_ are ${w}_{0i}={\gamma }_{i}/ \left( 1+\phi {\gamma }_{i} \right) + \left( \phi {\gamma }_{i} \left( {y}_{i}-{\gamma }_{i} \right) \right) /{ \left( 1+\phi {\gamma }_{i} \right) }^{2}$.

Second, the statistic *U* depends on the first derivate of the log-likelihood function. The formula for the *U* statistic depends on an assumption. Under the Poisson assumption, *U* is equal to ${U}_{Pois}= \frac{1}{2} \left( { \left( \mathbf{Y }-{g}^{-1} \left( \hat {\mu }+\mathbf{K}\hat {\alpha } \right) \right) }^{T}\mathbf{X}{\mathbf{X}}^{T} \left( \mathbf{Y }-{g}^{-1} \left( \hat {\mu }+\mathbf{K}\hat {\alpha } \right) \right) -tr \left( \hat {\mathbf{W}}\mathbf{X}{\mathbf{X}}^{T} \right) \right) $, while under the NB assumption, *U* is formulated as ${U}_{NB}= \frac{1}{2} \left( { \left( \mathbf{Y }-{g}^{-1} \left( \hat {\mu }+\mathbf{K}\hat {\alpha } \right) \right) }^{T}\hat {\Phi }\mathbf{X}{\mathbf{X}}^{T}\hat {\Phi } \right. \left. \left( \mathbf{Y }-{g}^{-1} \left( \hat {\mu }+\mathbf{K}\hat {\alpha } \right) \right) -tr \left( {\hat {\mathbf{W}}}_{\mathbf{ 0}}\mathbf{X}{\mathbf{X}}^{T} \right) \right) $. We estimate $\hat {\mu }$ and $\hat {\alpha }$ of the model under *H*_0_.

Third, the chi-square statistic is shown as ${\chi }^{2}=U\widetilde {I}{ \left( \hat {\alpha } \right) }^{-1}U$. The formula of the information matrix is as follows:

\begin{eqnarray*}\widetilde {I}={I}_{\tau \tau }-{I}_{\widetilde {\alpha }\tau }{I}_{\widetilde {\alpha }\widetilde {\alpha }}^{-1}{I}_{\widetilde {\alpha }\tau } \end{eqnarray*}

where ${I}_{\tau \tau }=E \left( \frac{\partial l}{\partial \tau } \frac{\partial l}{\partial \tau } \right) ,{I}_{\widetilde {\alpha }\tau }=E \left( \frac{\partial l}{\partial \widetilde {\alpha }} \frac{\partial l}{\partial \tau } \right) ,{I}_{\widetilde {\alpha }\widetilde {\alpha }}=E \left( \frac{\partial l}{\partial \widetilde {\alpha }} \frac{\partial l}{\partial \widetilde {\alpha }} \right) $, and $\widetilde {\alpha }= \left( \mu ,\alpha \right) $. Then, the formula is rewritten as follows:

\begin{eqnarray*}g \left( E \left( \mathbf{Y } \right) \right) =\widetilde {\mathbf{K}}\widetilde {\alpha }+\mathbf{X}\beta \end{eqnarray*}

where $\widetilde {\mathbf{K}}={ \left( {\widetilde {\mathbf{I}}}_{p},\ldots ,{\widetilde {\mathbf{I}}}_{p} \right) }^{T},{\widetilde {\mathbf{I}}}_{p}= \left( 1,{\mathbf{I}}_{p} \right) $.

Finally, suppose **A** = **K**^*T*^**K** with a diagonal element of *a*_*ii*_. *a*_*ii*_ consists of a vector, denoted as $\widetilde {\mathbf{a}}$. Let *κ*_2*i*_, *κ*_3*i*_ and *κ*_4*i*_ be the cumulants of **Y**_*i*_. With the assumption of an exponential family of distributions, the relationships between the three cumulants are as follows:

\begin{eqnarray*}\begin{array}{@{}c@{}} \displaystyle {\kappa }_{2i}=V \left( {\gamma }_{i} \right) \\ \displaystyle {\kappa }_{3i}={V}^{{^{\prime}}} \left( {\gamma }_{i} \right) V \left( {\gamma }_{i} \right) \\ \displaystyle {\kappa }_{3i}= \left( {V}^{{^{\prime\prime}}} \left( {\gamma }_{i} \right) V \left( {\gamma }_{i} \right) +{ \left( {V}^{{^{\prime}}} \left( {\gamma }_{i} \right) \right) }^{2} \right) V \left( {\gamma }_{i} \right) . \end{array} \end{eqnarray*}

Let **R** ∈ ℝ^*Np*×*Np*^ be a block diagonal matrix. In each block, the diagonal elements and non-diagonal elements are calculated as ${r}_{ii}^{j}={w}_{i}^{4}{\delta }_{i}^{-4}{\kappa }_{4i}+2{w}_{i}^{2}+{e}_{i}^{2}{\kappa }_{2i}-2{w}_{i}^{2}{\delta }_{i}^{-2}{e}_{i}{\kappa }_{3i}$ and ${r}_{i{i}^{{^{\prime}}}}^{j}=2{w}_{i}{w}_{{i}^{{^{\prime}}}},i\not = {i}^{{^{\prime}}}$, respectively. **C** ∈ ℝ^*Np*×*Np*^ is composed of a *p* diagonal matrix, *C*^*j*^, whose diagonal elements are ${c}_{ii}^{j}={w}_{i}^{4}{\delta }_{i}^{-4}{\kappa }_{3i}+2{w}_{i}^{2}+{e}_{i}^{2}{\kappa }_{2i}-2{w}_{i}^{2}{\delta }_{i}^{-2}{e}_{i}{\kappa }_{3i}$. Then, the elements of the information matrix are as follows:

\begin{eqnarray*}{I}_{\tau \tau }= \frac{1}{4} {\mathbf{J}}^{T} \left( \mathbf{ARA} \right) \mathbf{J}, {I}_{\widetilde {\alpha }\tau }= \frac{1}{2} \widetilde {\mathbf{K}}\mathbf{C}\widetilde {\mathbf{a}}, {I}_{\widetilde {\alpha }\widetilde {\alpha }}={\widetilde {\mathbf{K}}}^{T}\mathbf{W}\widetilde {\mathbf{K}} \end{eqnarray*}

where **J** is an *Np* vector, and its elements are 1. The difference in variances leads to different expressions for **R** and **C**. For the Poisson assumption, the diagonal and off-diagonal elements of **R** are ${r}_{ii}^{j}={\gamma }_{i}+2{\gamma }_{i}^{2}$ and ${r}_{i\widetilde {i}}=2{\gamma }_{i}{\gamma }_{\widetilde {i}},i\not = \widetilde {i}$, respectively. The diagonal elements of **C** are ${c}_{ii}^{j}={\gamma }_{i}$. For the NB assumption, the diagonal and off-diagonal elements of **R** are ${r}_{ii}^{j}= \frac{2{\gamma }_{i}^{3}{\phi }^{2}+3{\gamma }_{i}^{3}+4{\gamma }_{i}\phi +2{\gamma }_{i}^{2}+{\gamma }_{i}}{{ \left( 1+{\gamma }_{i}\phi \right) }^{3}} $ and ${r}_{i\widetilde {i}}=2 \frac{{\gamma }_{i}}{1+{\gamma }_{i}\phi } \frac{{\gamma }_{\widetilde {i}}}{1+{\gamma }_{\widetilde {i}}\phi } ,i\not = \widetilde {i}$, respectively. The diagonal elements of **C** are ${c}_{ii}^{j}= \frac{{\gamma }_{i}+{\gamma }_{i}^{2}\phi }{{ \left( 1+{\gamma }_{i}^{2}\phi \right) }^{2}} $.

### Empirical distribution of statistics

The permutation algorithm generates the empirical distribution. The two different assumptions cause different score statistics. For the Poisson assumption, the statistic is ${U}_{Pois}= \frac{1}{2} \left( { \left( \mathbf{Y }-{g}^{-1} \left( \hat {\mu }+\mathbf{K}\hat {\alpha } \right) \right) }^{T}\mathbf{X}{\mathbf{X}}^{T} \left( \mathbf{Y }-{g}^{-1} \left( \hat {\mu }+\mathbf{K}\hat {\alpha } \right) \right) -tr \left( \hat {\mathbf{W}}\mathbf{X}{\mathbf{X}}^{T} \right) \right) $. For the NB assumption, the statistic is equal to ${U}_{NB}= \frac{1}{2} ({ \left( \mathbf{Y }-{g}^{-1} \left( \hat {\mu }+\mathbf{K}\hat {\alpha } \right) \right) }^{T}\hat {\Phi }\mathbf{X}{\mathbf{X}}^{T}\hat {\Phi }(\mathbf{Y }-{g}^{-1}(\hat {\mu }+\mathbf{K}\hat {\alpha }))-tr({\hat {\mathbf{W}}}_{\mathbf{ 0}}\mathbf{X}{\mathbf{X}}^{T}))$. The hypothesis testing of *U* follows two steps: (a) construction of the empirical distribution by shuffling the label and (b) calculation of the *P* value of the original statistic *U*. The estimation of *μ* and *α* is similar to the above. To improve robustness, the default setting of the number of permutations is 5,000.

### Simulations

We perform simulations to examine the type I error and power of the proposed score statistics, *U*, for identifying differential expression under a range of scenarios. The parameter settings are based on the analysis of real data and references (Lehmann et al., 2011). We assume that the RNA-Seq data follow NB and Poisson distributions. Five parameters are involved: the variance of *α* (*s*), the variance of the disease effect (*l*), the number of isoforms (*p*), the logarithm of expression levels ($\exp \left( M \right) $) and the dispersion parameter (*d*). *s* shows the heterogeneity of the baseline abundance of each isoform. *l* refers to the heterogeneity of the disease effect. The other three parameters describe the characteristics of the expression levels. The variance of *α* varies from 0.25 to 1.75 in steps of 0.75. The variance of *β* varies from 0.20 to 1.00 in steps of 0.4. The number of isoforms can be 2, 4 or 8. *d* is set at 0.5 or 2. We let $\exp \left( M \right) $ be 2.5 or 5. The sample size is fixed at 40. In the type I error simulations, the effect size is set to zero (*l* = 0).

We compare our approach with three traditional algorithms: DESeq, edgeR and TSPM (Anders & Huber, 2010; Lehmann et al., 2011; Robinson, McCarthy & Smyth, 2010). The sum of isoforms is assumed to represent the expression level of one gene in these methods. Therefore, the loop number is the gene number in simulations. When *l* is equal to 0, the proportion of significant genes is the false positive rate.

### Real data analysis

We download the LUSC Batch 101 mRNA sequencing data from TCGA (Network, 2012). The sample size is 12 for the case-control traits. The number of genes is 20,533. Each sample has four files: *gene expression*, *normalized gene expression*, *isoform expression* and *normalized isoform expression*. We only used the *gene expression* and *isoform expression* datasets. These data are attached as File S1. To utilize the advantages of isoVCT, the candidate genes are the genes showing a higher expression level than the total sample, with isoforms that exhibit heterogeneous effect sizes. Finally, we select 6,134 genes.

### Software and algorithms

This algorithm is completed using the Microsoft R Open (v3.3.0; Microsoft Corp., Seattle, WA, USA). The functions *glmer* and *glmer.nb* in the *lme4* package fit the mixed models in our algorithm. The function *ginv* in the MASS package is employed to calculate the generalized inverse of the singular matrix. The packages *DESeq* and *edgeR* are both from Bioconductor. In consideration of the potential computing time of the simulations, the *doParallel* package is used for parallel computation.

This algorithm provides self-adaptation for real data analysis. If the fitness of the NB assumption fails or the dispersion parameter is close to one, the Poisson assumption is used. Due to the application of the idea of the variance component test, our method is referred to isoVCT. The distribution of the score test statistic is fitted via theoretical and empirical methods, which we term isoVCT-The and isoVCT-Emp, respectively. The R program of isoVCT and the simulation code are attached as File S2.

## Results

### Simulations with the NB assumption

**Figure 1:**
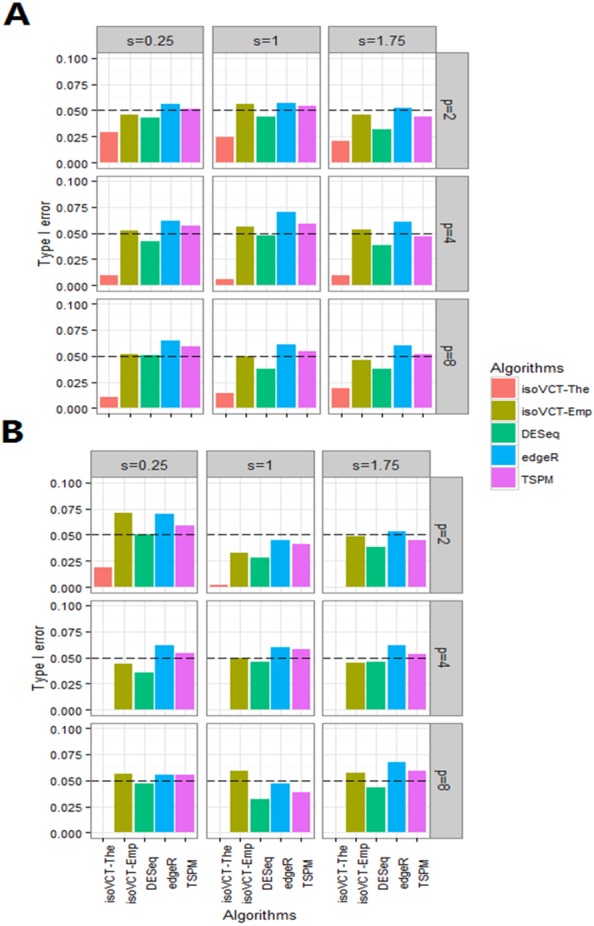
Plots of type I error of five algorithms in NB assumption. (A) The parameter setting is mu = 5 and phi = 2. (B) The parameter setting is mu = 5 and phi = 0.5.

The results for the empirical type I error for the NB assumption are shown in Table 1 and Fig. 1. *s*, *l* and *p* may be unrelated to type I error rates, but *ϕ* may show an inverse correlation to the type I error rate. isoVCT-The is exactly conservative in specific scenarios. The type I error rate of isoVCT-Emp is close to 0.05, which might mean that the permutation can fit the distribution of *U* in a small sample size setting. DESeq can control type I errors; however, the type I error rates of edgeR and TSPM are both dispersed, especially when *ϕ* = 0.5. Generally, isoVCT can control type I error rates around the nominal level for the NB assumption.

**Table 1:** The type I error rate of five algorithms in NB assumptions.

*M*	*s*	*p*	*ϕ* = 2					*ϕ* = 0.5				
			The	Emp	DESeq	edgeR	TSPM	The	Emp	DESeq	edgeR	TSPM
2.5	0.20	2	0.034	0.056	0.033	0.053	0.051	0.029	0.049	0.044	0.068	0.062
		4	0.024	0.049	0.041	0.056	0.053	0.031	0.051	0.033	0.051	0.050
		8	0.020	0.049	0.034	0.047	0.045	0.020	0.063	0.037	0.060	0.052
	0.60	2	0.022	0.053	0.041	0.059	0.057	0.013	0.039	0.047	0.058	0.052
		4	0.014	0.048	0.035	0.055	0.051	0.024	0.051	0.041	0.064	0.049
		8	0.010	0.056	0.037	0.057	0.050	0.013	0.047	0.036	0.045	0.043
	1.00	2	0.024	0.049	0.033	0.050	0.047	0.032	0.050	0.034	0.050	0.059
		4	0.019	0.048	0.041	0.060	0.052	0.019	0.058	0.038	0.057	0.054
		8	0.007	0.042	0.036	0.061	0.055	0.000	0.045	0.029	0.051	0.045
5.0	0.20	2	0.029	0.046	0.043	0.056	0.051	0.019	0.071	0.050	0.070	0.059
		4	0.010	0.052	0.042	0.062	0.057	0.000	0.044	0.036	0.062	0.054
		8	0.011	0.052	0.051	0.065	0.059	0.000	0.056	0.047	0.055	0.055
	0.60	2	0.024	0.056	0.044	0.057	0.054	0.002	0.033	0.028	0.045	0.041
		4	0.006	0.056	0.048	0.070	0.059	0.000	0.050	0.046	0.060	0.058
		8	0.014	0.050	0.038	0.061	0.054	0.000	0.059	0.032	0.047	0.039
	1.00	2	0.021	0.046	0.032	0.052	0.044	0.000	0.049	0.038	0.053	0.045
		4	0.010	0.053	0.038	0.061	0.047	0.000	0.045	0.046	0.062	0.053
		8	0.019	0.046	0.038	0.060	0.052	0.000	0.057	0.043	0.067	0.059

**Figure 2:**
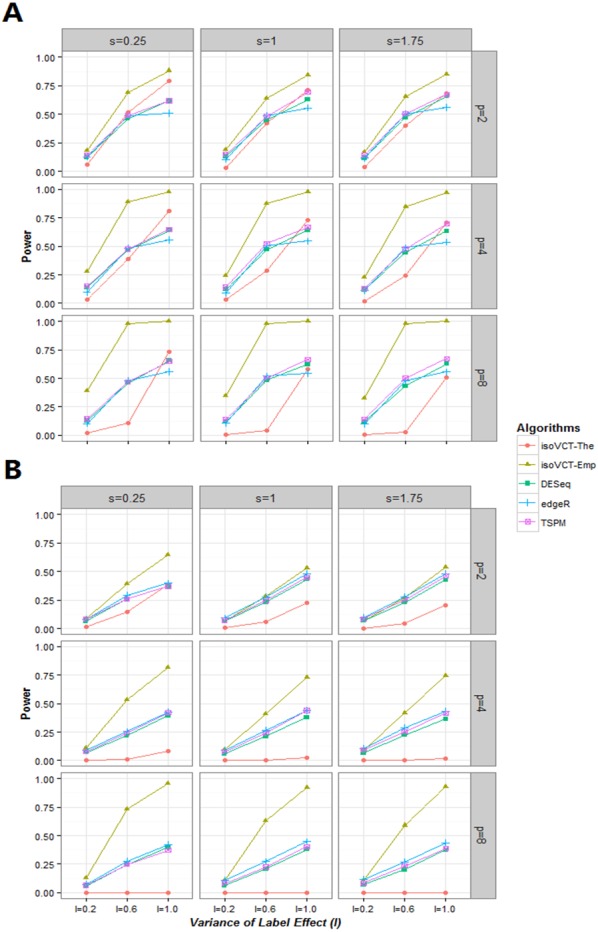
Plots of power of five algorithms in NB assumption. (A) The parameter setting is mu = 5 and phi = 2. (B) The parameter setting is mu = 5 and phi = 0.5.

The results regarding empirical power for the NB assumption are shown in Tables 2, 3 and Fig. 2. Three findings of this analysis are as follows: (a) *M* exhibits a positive correlation with power; (b) *p* and *s* exhibit a negative correlation with power; and (c) *l* also exhibits a positive correlation with power, but its increase is much sharper than those of *p* and *M*. Our results show that isoVCT-Emp is more advantageous than traditional methods, especially when the effect size is small and the effect heterogeneity is large.

**Table 2:** The power of five algorithms in NB assumptions (exp(*M*) = 5).

*l*	*s*	*p*	*ϕ* = 2					*ϕ* = 0.5				
			The	Emp	DESeq	edgeR	TSPM	The	Emp	DESeq	edgeR	TSPM
0.2	0.25	2	0.057	0.182	0.124	0.127	0.141	0.018	0.089	0.065	0.080	0.083
		4	0.029	0.277	0.139	0.096	0.151	0.000	0.109	0.071	0.087	0.079
		8	0.020	0.391	0.130	0.103	0.144	0.000	0.129	0.058	0.074	0.065
	1.00	2	0.031	0.186	0.130	0.104	0.148	0.005	0.065	0.063	0.091	0.073
		4	0.029	0.243	0.123	0.090	0.142	0.000	0.095	0.059	0.092	0.077
		8	0.010	0.349	0.119	0.105	0.140	0.000	0.105	0.065	0.108	0.080
	1.75	2	0.039	0.167	0.116	0.116	0.141	0.002	0.069	0.069	0.095	0.085
		4	0.021	0.228	0.119	0.109	0.133	0.000	0.094	0.067	0.107	0.093
		8	0.010	0.323	0.120	0.100	0.141	0.000	0.106	0.070	0.119	0.083
0.6	0.25	2	0.518	0.691	0.461	0.490	0.490	0.144	0.394	0.258	0.290	0.260
		4	0.390	0.890	0.465	0.482	0.475	0.011	0.530	0.219	0.256	0.242
		8	0.107	0.980	0.459	0.482	0.468	0.000	0.734	0.244	0.275	0.247
	1.00	2	0.420	0.640	0.447	0.488	0.481	0.059	0.285	0.233	0.278	0.247
		4	0.286	0.876	0.470	0.506	0.525	0.002	0.410	0.212	0.265	0.241
		8	0.045	0.981	0.483	0.520	0.503	0.000	0.631	0.209	0.273	0.225
	1.75	2	0.400	0.652	0.472	0.503	0.503	0.043	0.269	0.227	0.279	0.248
		4	0.241	0.847	0.443	0.487	0.475	0.001	0.417	0.224	0.289	0.252
		8	0.030	0.978	0.432	0.480	0.502	0.000	0.591	0.202	0.268	0.233
1.0	0.25	2	0.792	0.882	0.616	0.508	0.617	0.383	0.645	0.367	0.396	0.371
		4	0.809	0.979	0.636	0.555	0.648	0.083	0.814	0.393	0.425	0.418
		8	0.730	0.999	0.656	0.558	0.648	0.000	0.958	0.397	0.422	0.372
	1.00	2	0.709	0.839	0.628	0.555	0.691	0.224	0.529	0.431	0.481	0.447
		4	0.729	0.975	0.641	0.546	0.666	0.027	0.727	0.378	0.439	0.439
		8	0.579	1.000	0.622	0.544	0.665	0.000	0.920	0.378	0.446	0.405
	1.75	2	0.683	0.849	0.656	0.558	0.673	0.203	0.539	0.427	0.482	0.459
		4	0.710	0.968	0.633	0.536	0.694	0.015	0.744	0.364	0.433	0.415
		8	0.505	1.000	0.627	0.557	0.673	0.000	0.928	0.374	0.437	0.386

**Table 3:** The power of five algorithms in NB assumptions (exp(*M*) = 2.5).

*l*	*s*	*p*	*ϕ* = 2					*ϕ* = 0.5				
			The	Emp	DESeq	edgeR	TSPM	The	Emp	DESeq	edgeR	TSPM
0.2	0.25	2	0.057	0.181	0.110	0.086	0.130	0.014	0.081	0.052	0.071	0.078
		4	0.022	0.242	0.113	0.110	0.134	0.000	0.104	0.060	0.084	0.080
		8	0.019	0.365	0.111	0.096	0.124	0.000	0.129	0.063	0.084	0.077
	1.00	2	0.031	0.143	0.121	0.094	0.142	0.006	0.079	0.072	0.094	0.092
		4	0.027	0.208	0.115	0.103	0.141	0.000	0.099	0.069	0.100	0.089
		8	0.010	0.309	0.105	0.109	0.127	0.000	0.101	0.058	0.098	0.074
	1.75	2	0.029	0.144	0.111	0.090	0.127	0.007	0.073	0.068	0.100	0.092
		4	0.020	0.203	0.107	0.091	0.137	0.000	0.086	0.072	0.107	0.085
		8	0.010	0.285	0.121	0.103	0.146	0.000	0.087	0.066	0.103	0.073
0.6	0.25	2	0.527	0.705	0.429	0.460	0.452	0.152	0.392	0.210	0.261	0.236
		4	0.325	0.864	0.445	0.470	0.452	0.011	0.522	0.210	0.239	0.220
		8	0.069	0.978	0.459	0.484	0.465	0.000	0.735	0.218	0.255	0.235
	1.00	2	0.357	0.592	0.448	0.473	0.492	0.060	0.300	0.231	0.281	0.272
		4	0.208	0.831	0.443	0.478	0.484	0.002	0.400	0.217	0.276	0.245
		8	0.020	0.970	0.455	0.494	0.473	0.000	0.614	0.194	0.247	0.217
	1.75	2	0.330	0.584	0.449	0.488	0.471	0.034	0.254	0.200	0.245	0.240
		4	0.158	0.786	0.445	0.487	0.479	0.001	0.383	0.201	0.259	0.219
		8	0.022	0.957	0.441	0.482	0.504	0.000	0.572	0.200	0.283	0.237
1.0	0.25	2	0.760	0.857	0.592	0.512	0.620	0.319	0.625	0.352	0.404	0.386
		4	0.773	0.974	0.630	0.525	0.643	0.067	0.822	0.368	0.396	0.375
		8	0.589	0.999	0.617	0.532	0.610	0.000	0.957	0.385	0.410	0.360
	1.00	2	0.663	0.820	0.650	0.524	0.660	0.180	0.505	0.382	0.432	0.417
		4	0.623	0.958	0.610	0.536	0.623	0.024	0.717	0.407	0.463	0.423
		8	0.415	0.997	0.634	0.537	0.666	0.000	0.933	0.368	0.442	0.391
	1.75	2	0.593	0.791	0.636	0.539	0.658	0.145	0.499	0.389	0.447	0.443
		4	0.595	0.9520	0.628	0.518	0.663	0.013	0.716	0.396	0.446	0.426
		8	0.345	0.9955	0.623	0.569	0.668	0.000	0.914	0.393	0.454	0.405

### Simulations with the Poisson assumption

The results of the type I error and empirical power under the Poisson assumption are shown in File S3.

### Real data analysis

**Figure 3:**
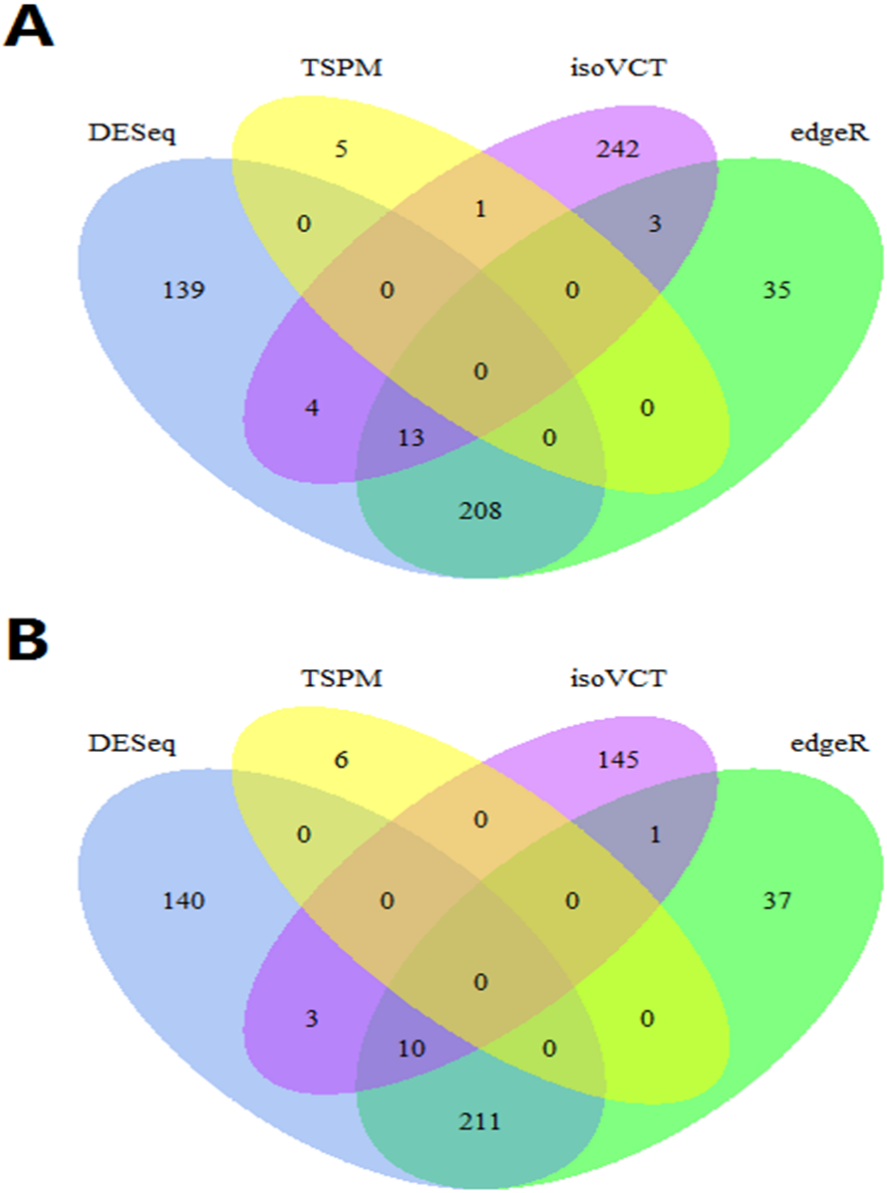
Venn diagrams of different methods in real data analysis. (A) Venn diagram of DESeq, edgeR, TSPM and isoVCT-Emp of non-normalized data; (B) Venn diagram of DESeq, edgeR, TSPM and isoVCT-The of non-normalized data.

The results of real data analysis are shown in Fig. 3. Only 4,422 genes are fit to the GLMM. The three traditional algorithms, DESeq, edgeR and TSPM, define 364, 259 and six DE genes, respectively. The intersection of DESeq and edgeR is 221 genes, representing a high proportion of the DE genes that these algorithms define. TSPM is almost ineffective for these genes.

The results of isoVCT are as follows. First, isoVCT-Emp defines 263 DE genes; of these genes, isoVCT-Emp specifically selected 242 DE genes, and only a small portion of these genes is also found by the other algorithms. Second, the DE genes identified by isoVCT-Emp included those that are identified by isoVCT-The. In general, isoVCT is superior at selecting heterogeneous genes.

## Discussion

Here, we propose isoVCT, a variance component score test for testing the coefficients of covariates to select DE genes. Simulations and real data analysis both suggest that this method is advantageous in selecting weak effects or heterogeneous genes. The results of the simulations are discussed from the following two aspects. First, isoVCT controls type I errors in each setting under any of the distribution assumptions. The type I error rate of the empirical distribution is almost controlled. However, the type I error rate of isoVCT-The is strictly controlled, which is likely caused by an inaccurate estimation of the *U* statistic in the small sample size setting. The type I error rates of edgeR and TSPM increase in some settings, especially with the NB assumption (Yang et al., 2015). Second, the power of isoVCT is higher than that of other methods; furthermore, the strength is evident for the small *l* and NB assumption. isoVCT-Emp is superior to other methods in each setting,especially for *l* = 0.6. The small sample size setting may cause the inverse proportion between *p* and the power of isoVCT. Nevertheless, the powers of five algorithms are high for the Poisson assumption.

In real data analysis, the Venn diagram of DE genes directly indicates the relationships among the four algorithms. The fact that DESeq and edgeR use the same distribution assumption and the same idea for testing likely results in the number of intersections representing a majority of the DE genes identified by these algorithms. TSPM defines the smallest number of DE genes. However, the use of different ideas leads to the different result of isoVCT. isoVCT-Emp specifically defines 242 heterogeneous or small effect size genes. For example, *CASP7* and *STAT6* are related to LUSC (Dubey & Saini, 2015; Lee et al., 2009).

Furthermore, isoVCT exhibits three innovations. First, isoVCT derives the empirical and theoretical distribution of the variance component score statistics in the framework of a GLMM and evaluates the performance of score statistics. Second, the random effect α represents the correlation of isoforms in the framework of the GLMM. Third, isoVCT further verifies the effectiveness of the set test in RNA-Seq data and supplies a new view of biological functions with isoform information.

However, isoVCT has some limitations. In the derivation of the score statistic, the random term *α* is regarded as a fixed parameter, which may cause isoVCT-The to be quite conservative. For the NB assumption, the power of isoVCT is very low in some settings, because the estimation of the dispersion parameter may be biased. The methods of weighted likelihood and quasi-likelihood likely overcome this drawback in estimating the NB mixed model (Lund et al., 2012).

In conclusion, isoVCT, a supplement to DESeq or edgeR, is powerful and robust at selecting small effect and heterogeneous genes.

##  Supplemental Information

10.7717/peerj.3797/supp-1 File S1 Real data

10.7717/peerj.3797/supp-2 File S2 R code

10.7717/peerj.3797/supp-3 File S3 Summary of simulations in Poisson assumption

## Additional Information and Declarations

### Data Availability

The following information was supplied regarding data availability: The raw data has been supplied as Supplemental Files.
