# Supplementary material for: Variance component testing for identifying differentially expressed genes in RNA-seq data
Source: PeerJ. 2017 Sep 8;5:e3797. doi: 10.7717/peerj.3797 (PMC5592911; doi:10.7717/peerj.3797)
Supplement: File S3 [file peerj-05-3797-s003.docx]

**Supplementary file 3 Summary of simulations in Poisson assumption**

The results of the empirical type I error for Poisson assumption are shown in **Table 1**. *M* and *s* are likely unrelated to type I errors in every algorithm. However, the increase in *p* possibly decreases the type I error rate when *M*=2.5. The type I error rate of isoVCT is similar to that for the NB assumption. DESeq and edgeR are conservative in multiple settings; however, the type I error rate of TSPM is dispersed.

The empirical power results for the Poisson assumption are shown in **Table 2**. In contrast to the situation for the NB assumption, we find three results: a) *p* and *M* have a positive correlation with power; b) *s* has a negative correlation with power; and c) *l* also has a positive correlation with power, but its increase is much sharper than that of *p* and *M*. The other findings are similar to those for the NB assumption.

**Table 1** The type I error rates of five algorithms in Poisson assumption

| *M* | *s* | *p* | Algorithms | | | | |
| --- | --- | --- | --- | --- | --- | --- | --- |
|  |  |  | The | Emp | DESeq | edgeR | TSPM |
| 2.5 | 0.20 | 2 | 0.037 | 0.039 | 0.034 | 0.041 | 0.051 |
|  |  | 4 | 0.022 | 0.048 | 0.032 | 0.037 | 0.047 |
|  |  | 8 | 0.013 | 0.051 | 0.041 | 0.047 | 0.055 |
|  | 0.60 | 2 | 0.046 | 0.045 | 0.027 | 0.032 | 0.045 |
|  |  | 4 | 0.035 | 0.052 | 0.033 | 0.036 | 0.055 |
|  |  | 8 | 0.028 | 0.046 | 0.038 | 0.042 | 0.057 |
|  | 1.00 | 2 | 0.043 | 0.045 | 0.031 | 0.037 | 0.053 |
|  |  | 4 | 0.036 | 0.052 | 0.032 | 0.037 | 0.047 |
|  |  | 8 | 0.027 | 0.058 | 0.038 | 0.046 | 0.055 |
| 5.0 | 0.20 | 2 | 0.047 | 0.050 | 0.037 | 0.043 | 0.057 |
|  |  | 4 | 0.033 | 0.048 | 0.036 | 0.043 | 0.054 |
|  |  | 8 | 0.021 | 0.046 | 0.030 | 0.033 | 0.040 |
|  | 0.60 | 2 | 0.044 | 0.046 | 0.040 | 0.043 | 0.054 |
|  |  | 4 | 0.038 | 0.055 | 0.029 | 0.037 | 0.043 |
|  |  | 8 | 0.025 | 0.059 | 0.040 | 0.047 | 0.061 |
|  | 1.00 | 2 | 0.036 | 0.052 | 0.040 | 0.044 | 0.058 |
|  |  | 4 | 0.034 | 0.051 | 0.044 | 0.053 | 0.058 |
|  |  | 8 | 0.020 | 0.057 | 0.043 | 0.048 | 0.059 |

**Table 2** The power of five algorithms in Poisson assumption

| *l* | *s* | *p* | $\exp\left(M\right)=2.5$ | | | | |  | $\exp\left(M\right)=5$ | | | | |
| --- | --- | --- | --- | --- | --- | --- | --- | --- | --- | --- | --- | --- | --- |
|  |  |  | The | Emp | DESeq | edgeR | TSPM |  | The | Emp | DESeq | edgeR | TSPM |
| 0.2 | 0.25 | 2 | 0.212 | 0.378 | 0.254 | 0.274 | 0.293 |  | 0.829 | 0.892 | 0.723 | 0.732 | 0.735 |
|  |  | 4 | 0.134 | 0.589 | 0.266 | 0.281 | 0.311 |  | 0.909 | 0.988 | 0.727 | 0.736 | 0.740 |
|  |  | 8 | 0.027 | 0.808 | 0.287 | 0.301 | 0.326 |  | 0.944 | 0.999 | 0.721 | 0.732 | 0.739 |
|  | 1.00 | 2 | 0.279 | 0.421 | 0.340 | 0.359 | 0.377 |  | 0.808 | 0.864 | 0.739 | 0.747 | 0.762 |
|  |  | 4 | 0.260 | 0.620 | 0.401 | 0.414 | 0.420 |  | 0.876 | 0.971 | 0.777 | 0.785 | 0.786 |
|  |  | 8 | 0.193 | 0.800 | 0.451 | 0.465 | 0.478 |  | 0.939 | 0.999 | 0.810 | 0.814 | 0.820 |
|  | 1.75 | 2 | 0.352 | 0.483 | 0.421 | 0.448 | 0.464 |  | 0.763 | 0.831 | 0.757 | 0.771 | 0.780 |
|  |  | 4 | 0.411 | 0.673 | 0.528 | 0.544 | 0.549 |  | 0.887 | 0.961 | 0.842 | 0.844 | 0.853 |
|  |  | 8 | 0.438 | 0.865 | 0.616 | 0.627 | 0.655 |  | 0.930 | 0.994 | 0.877 | 0.879 | 0.886 |
| 0.6 | 0.25 | 2 | 0.668 | 0.731 | 0.581 | 0.599 | 0.624 |  | 0.977 | 0.985 | 0.900 | 0.909 | 0.911 |
|  |  | 4 | 0.803 | 0.866 | 0.676 | 0.685 | 0.690 |  | 0.998 | 1.000 | 0.905 | 0.909 | 0.917 |
|  |  | 8 | 0.681 | 0.917 | 0.566 | 0.586 | 0.598 |  | 1.000 | 1.000 | 0.921 | 0.924 | 0.924 |
|  | 1.00 | 2 | 0.845 | 0.977 | 0.683 | 0.692 | 0.715 |  | 0.965 | 0.979 | 0.921 | 0.923 | 0.933 |
|  |  | 4 | 0.678 | 0.987 | 0.575 | 0.594 | 0.613 |  | 0.990 | 0.998 | 0.923 | 0.928 | 0.940 |
|  |  | 8 | 0.905 | 1.000 | 0.681 | 0.691 | 0.693 |  | 1.000 | 1.000 | 0.934 | 0.936 | 0.942 |
|  | 1.75 | 2 | 0.620 | 0.687 | 0.601 | 0.623 | 0.639 |  | 0.952 | 0.972 | 0.914 | 0.919 | 0.921 |
|  |  | 4 | 0.733 | 0.822 | 0.705 | 0.714 | 0.754 |  | 0.992 | 0.998 | 0.952 | 0.953 | 0.959 |
|  |  | 8 | 0.662 | 0.889 | 0.650 | 0.662 | 0.672 |  | 0.996 | 1.000 | 0.963 | 0.963 | 0.966 |
| 1.0 | 0.25 | 2 | 0.908 | 0.945 | 0.829 | 0.833 | 0.849 |  | 0.994 | 0.997 | 0.946 | 0.947 | 0.962 |
|  |  | 4 | 0.969 | 0.996 | 0.813 | 0.818 | 0.861 |  | 1.000 | 1.000 | 0.950 | 0.953 | 0.950 |
|  |  | 8 | 0.995 | 1.000 | 0.841 | 0.841 | 0.842 |  | 1.000 | 1.000 | 0.951 | 0.951 | 0.963 |
|  | 1.00 | 2 | 0.863 | 0.904 | 0.818 | 0.824 | 0.861 |  | 0.988 | 0.993 | 0.948 | 0.952 | 0.957 |
|  |  | 4 | 0.939 | 0.988 | 0.861 | 0.862 | 0.874 |  | 1.000 | 1.000 | 0.953 | 0.957 | 0.964 |
|  |  | 8 | 0.988 | 1.000 | 0.893 | 0.895 | 0.902 |  | 1.000 | 1.000 | 0.966 | 0.966 | 0.967 |
|  | 1.75 | 2 | 0.856 | 0.900 | 0.816 | 0.825 | 0.864 |  | 0.971 | 0.982 | 0.949 | 0.952 | 0.967 |
|  |  | 4 | 0.939 | 0.981 | 0.876 | 0.880 | 0.918 |  | 0.997 | 0.999 | 0.972 | 0.971 | 0.978 |
|  |  | 8 | 0.971 | 0.999 | 0.923 | 0.925 | 0.937 |  | 1.000 | 1.000 | 0.981 | 0.977 | 0.983 |
